# The Interplay Between Iron Metabolism and Insulin Resistance: A Key Factor in Optimizing Obesity Management in Children and Adolescents

**DOI:** 10.3390/nu17071211

**Published:** 2025-03-30

**Authors:** Valeria Calcaterra, Hellas Cena, Federica Bolpagni, Silvia Taranto, Alessandra Vincenti, Nagaia Madini, Marianna Diotti, Antonia Quatrale, Gianvincenzo Zuccotti

**Affiliations:** 1 Department of Internal Medicine and Therapeutics, University of Pavia, 27100 Pavia, Italy; valeria.calcaterra@unipv.it; 2 Pediatric Department, Buzzi Children’s Hospital, 20154 Milano, Italy; silvia.taranto@unimi.it (S.T.); nagaia.madini@unipv.it (N.M.); marianna.diotti@unimi.it (M.D.); antonia.quatrale@unimi.it (A.Q.); gianvincenzo.zuccotti@unimi.it (G.Z.); 3 Laboratory of Dietetics and Clinical Nutrition, Department of Public Health, Experimental and Forensic Medicine, University of Pavia, 27100 Pavia, Italy; 4 Clinical Nutrition and Dietetics Unit, ICS Maugeri IRCCS, 27100 Pavia, Italy; federica.bolpagni@community.unipa.it (F.B.); alessandra.vincenti@unipv.it (A.V.); 5 Department of Biomedical and Clinical Science, University of Milano, 20157 Milano, Italy

**Keywords:** childhood obesity, iron deficiency, insulin resistance, nutritional strategies, weight loss

## Abstract

Iron plays a vital role in insulin signaling, regulating molecular mechanisms that influence cellular insulin responses. This review explores the link between iron metabolism and insulin resistance (IR) in children and adolescents with obesity. A connection between iron metabolism, iron deficiency (ID), and IR is well-documented, but further longitudinal studies are needed to better understand how iron metabolism influences insulin resistance during childhood and adolescence. This connection warrants attention due to its significant public health implications, as optimizing obesity management could help prevent both ID and metabolic complications in children. Current evidence does not suggest that dietary factors are primary contributors to ID in children. However, there is scientific evidence that weight reduction can restore iron homeostasis in people with obesity. Therefore, efforts should focus on improving dietary habits, increasing awareness of iron’s importance, and implementing strategies to address both ID and obesity.

## 1. Introduction

Over the past forty years, the prevalence of childhood obesity has doubled among children aged 2–4 and increased eightfold among those aged 5–19 worldwide, reaching a rate of 18.5% among children and adolescents aged 2–19 and affecting 13.7 million individuals [1,2]. Childhood obesity is associated with numerous metabolic, inflammatory, and systemic complications. Hypertrophic adipose tissue releases adipokines and promotes chronic low-grade inflammation, which contributes to insulin resistance (IR), dyslipidemia, and hypertension. This inflammatory state is linked to an increased risk of autoimmune diseases, cancer, and endocrine disorders such as polycystic ovary syndrome and subclinical hypothyroidism. Additionally, pediatric obesity is associated with nonalcoholic fatty liver disease (NAFLD), gastrointestinal issues, respiratory disorders (asthma, obstructive sleep apnea syndrome), micronutrient deficiencies, and musculoskeletal conditions. Mental health is also affected, with a higher incidence of social isolation, bullying, and low self-esteem [3,4]. Approximately 75% of children with obesity remain obese in adulthood, which increases their risk of metabolic and cardiovascular morbidity and mortality [5,6,7,8].

The global obesity epidemic is caused by a multitude of interrelated genetic, epigenetic, and environmental variables. Major contributing factors include the imbalance in dietary intake, particularly with regard to energy-dense foods and sugar-filled beverages, the decreased opportunities for physical activity brought about by urbanization, and the growing use of modern media during free time [3,9].

Despite the increase in food intake, micronutrient deficiencies are very common in pediatric patients with obesity, particularly involving metals (iron, copper, zinc), vitamins (A, B, C, D, E), and folic acid [10].

Specifically, the literature delineates an association between overweight/obesity and iron deficiency (ID) among children and adolescents [11,12]. Indeed, children living with obesity appear to have a higher prevalence of ID compared to those without obesity [13]. On the other hand, there is scientific evidence that a decrease in body weight can restore iron homeostasis in people with obesity [14].

Additionally, recent research suggests that ID is associated with IR. Iron plays a crucial role in insulin signaling, influencing molecular regulatory mechanisms that affect cellular insulin responses. Furthermore, the balance between insulin and glucagon, which is vital for maintaining hepatic glucose production and systemic glucose levels, may be modulated by specific trace elements, including iron [15].

In this narrative review, we examined the interplay between iron metabolism and IR among children and adolescents with obesity. This connection warrants attention due to its significant public health implications, particularly in optimizing obesity management to prevent ID and metabolic complications in the pediatric population [14]. Efforts should focus on improving dietary habits, increasing awareness of iron’s importance, and implementing programs that address both ID and obesity [13].

## 2. Methods

Research was conducted to identify the relevant literature published in the last 10 years (2015–2025). The PubMed and Scopus databases were used. The following keywords (alone and/or in combination) were used for the research: pediatric obesity, insulin resistance, metabolic syndrome, iron metabolism, iron deficiency prevention, diet, nutritional management, and diet management. Moreover, the following MESH terms were used: Pediatric Obesity, Iron Metabolism Disorders, Insulin Resistance, and Nutrition Therapy.

The inclusion criteria adopted were original papers, clinical trials, meta-analyses, and reviews. Letters, single case reports, brief reports, and commentaries were excluded. Only human studies specifically involving participants younger than 18 years were included, and only English-language publications were considered.

A total of 1904 citations were identified (PubMed = 596, Scopus = 1308). After screening, 626 citations were excluded because they were duplicates, they were not original papers, clinical trials, meta-analyses, or reviews, they were in a language other than English, or they did not involve human participants. The authors assessed the titles and abstracts of the available literature (n = 1278), screened the full texts of potentially relevant articles (n = 272), and reviewed, analyzed, and discussed relevant full texts (n = 98).

**Figure 1:**
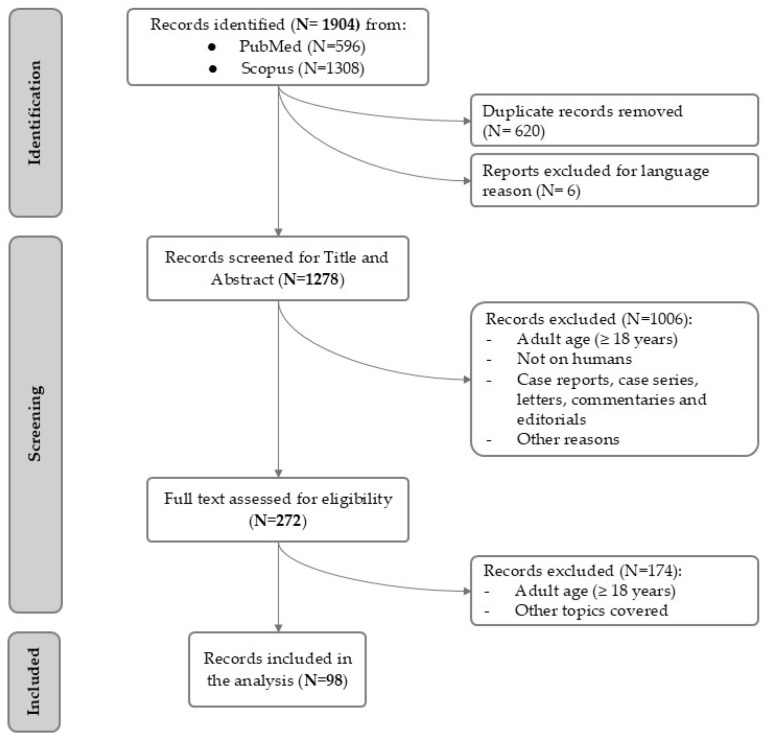
Search process of studies on iron deficiency, insulin resistance, and nutritional strategies to improve iron status in children and adolescents with obesity.

The search process is reported as a flow diagram in Figure 1.

## 3. Iron Deficiency and Insulin Resistance in Pediatric Obesity

### 3.1. Iron Deficiency in Pediatric Obesity

A major public health concern is ID, particularly in children and adolescents, whose fast growth and development necessitate increased iron requirements.

In a review of forty-eight articles, including observational studies, case reports, and interventional studies on the association between iron status, inflammatory markers, and iron intake in relation to weight status in children and adolescents, published in February 2016, observational studies conducted in high- and middle-income countries reported that the prevalence and risk of ID are significantly higher in children and adolescents with overweight and obesity compared to their normal-weight peers [16].

In a recent meta-analysis of 42 studies, including one cohort study, 29 cross-sectional studies, and 12 case–control studies, researchers compared 16,633 children living with obesity to 32,573 children without obesity. Among these, 16 studies focused on the association between iron deficiency (ID) and obesity, involving 3147 children with obesity and 17,802 children without obesity. The pooled prevalence of ID among children living with obesity was 20.07% (95% CI: 14.98, 25.16), while it was 16.10% (95% CI: 11.82, 20.38) among children without obesity. The pooled odds ratio (95% CI) was 1.64 (1.22, 2.21), *p* = 0.001 [13].

A systematic review and meta-analysis conducted in April 2024 analyzed 83 observational studies involving 190,443 children and adolescents with diagnosed undernutrition or overnutrition from 44 countries. The analysis synthesized data from seven studies, revealing that overnutrition (overweight and obesity) significantly increased the odds of iron deficiency (ID) (OR: 1.51, 95% CI: 1.20 to 1.82, *p* < 0.0001, *I*^2^ = 40.7%). Children with obesity had higher odds of ID (OR: 1.88, 95% CI: 1.33 to 2.43, *p* < 0.0001, *I*^2^ = 20.6%) compared to those with overweight (OR: 1.31, 95% CI: 0.98 to 1.64, *p* < 0.0001, *I*^2^ = 40.5%), although the difference between these groups was not statistically significant (*p* = 0.08) [17].

This result was consistent with a quantitative meta-analysis conducted in September 2015, which analyzed 26 cross-sectional and case–control studies, comprising 13,393 individuals with overweight/obesity and 26,621 non-overweight participants. In the subtype analysis, considering subjects aged below 18 years, the risk of ID in subjects with overweight/obesity versus non-overweight subjects was significantly higher (ES (95% CI) 1.78 (1.37, 2.30), *I*^2^ 76.5%, *p* value 0.025, 7 studies) [18].

Several other studies have supported the notion that childhood and teenage obesity is strongly linked to iron deficiency [19,20,21,22,23,24].

Correcting ID in the pediatric population could be of major interest because it is commonly accepted that ID can have a negative impact on cognitive function. Specifically, cognitive problems associated with ID are related to attention span, intellect, and sensory perception. Lower scores in language, motor skills, and environmental sound perception are linked to chronic ID [10,14,25,26].

### 3.2. Insulin Resistance in Pediatric Obesity

Obesity represents one of the major risk factors for IR during childhood and adolescence; a third of this population may also have glucose intolerance and relative beta-cell failure [27]. Teenagers are more likely than children to have IR, suggesting that puberty influences metabolic status. For example, IR is half as common at age 8 compared to age 15. IR is more common in men than in women and in white individuals and Hispanics than African Americans [28].

The first systematic review and meta-analysis to assess the IR status in adolescents with obesity aged 12–18 years, without concomitant diseases or syndromes, was published in 2017 and included 31 studies. The meta-analysis found significantly higher circulating fasting insulin and *C*-peptide levels, as well as higher Homeostasis Model Assessment of Insulin Resistance (HOMA-IR) values, in adolescents with obesity compared with those without obesity [29].

A recent study was conducted in the Department of Children’s Health Care and Endocrinology at the Children’s Hospital of Nanjing Medical University. A total of 88 normal-weight children and 171 children with obesity/overweight, aged between 6 and 14 years, were recruited. The prevalence of IR among these children and adolescents was 54%, while in the healthy counterparts it was 11%, showing a statistically significant difference [30].

A cross-sectional study was conducted among 150 children and adolescents with overweight and obesity, aged <18 years, who were referred to the Endocrinology Clinic of Imam Ali Hospital (Iran) in 2020. The results showed a significant correlation between HOMA-IR and insulin levels, as well as anthropometric indices; in particular, IR was positively associated with weight, waist-to-height ratio, BMI, and wrist circumference [31].

These results are consistent with evidence from a cross-sectional data analysis carried out in 2017–2018, which included a sample of 127 adolescents (70% girls) between 11 and 17 years of age. The IR group exhibited significantly higher values for waist circumference, waist-to-height ratio, android fat mass, android/gynoid ratio, visceral adipose tissue, glucose, and insulin (all *p* < 0.05) than the insulin-sensitive (IS) group [32].

### 3.3. Association Between Iron Deficiency and Insulin Resistance in Pediatric Obesity

Alterations in iron homeostasis have been linked to IR; however, research on this association in pediatric populations remains limited and yields conflicting results. As mentioned previously, pediatric obesity is strongly associated with the onset of multiple comorbidities, including nonalcoholic fatty liver disease, dyslipidemia, type 2 diabetes mellitus, acquired cardiovascular disorders, chronic inflammation, and anemia, among others. Within this intricate network of comorbidities and pathological conditions, obesity has been significantly connected to major disruptions in iron homeostasis. Although iron is the second most prevalent metal on Earth, its bioavailability is limited by its propensity to form highly insoluble oxides, making ID the most widespread nutritional disorder [33]. Low iron levels in children with overweight and obesity have been linked to lipid metabolism alterations, systemic inflammation, and IR [34]. Similar findings have been observed in adults. The mechanisms underlying low iron levels in obesity remain unclear. One hypothesis is that children and adolescents with obesity are at higher risk of ID due to unbalanced diets. Another possibility is that increased iron needs, driven by larger blood volume and higher basal losses, contribute to this deficiency. However, the most probable explanation is iron sequestration via an inflammation-mediated pathway [35,36]. Indeed iron metabolism is homeostatically regulated by hepcidin, a peptide whose expression is increased in chronic inflammatory states [37], such as obesity [38]. Hepcidin induces a decrease in iron uptake in the duodenum, the release of iron from macrophages, and the exit of stored iron from hepatocytes [33].

Markers of iron homeostasis are linked to IR in adults. Ferritin, an acute-phase reactant, rises in obesity alongside inflammation, complicating its role as a reliable indicator of iron stores. Transferrin, which transports iron in circulation, also increases with higher iron demand and inflammation. Soluble transferrin receptors (sTfR) regulate cellular iron uptake and are primarily influenced by ID rather than inflammation. In children and adolescents, there are still few studies on the relationship between iron metabolism and insulin resistance, but emerging evidence suggests that iron dysregulation may be similarly influential in pediatric populations. Klisic et al. found that sTfR levels correlate independently with HOMA-IR, while elevated ferritin and adipokines are associated with higher HOMA-IR in adolescent girls [39]. Similarly, Suàrez Ortégon et al. have shown a positive correlation between ferritin levels and increased insulin resistance in both children and adolescents [40]. Moschonis et al. found that body fat percentage and visceral fat mass were positively linked to ID in schoolchildren aged 9–13 years, suggesting that these associations might result from chronic inflammation due to excess adiposity [41]. This relationship is significant because obesity is the primary modifiable risk factor for insulin resistance among children and adolescents, and insulin resistance remains the most prevalent metabolic disorder associated with obesity [42]. In a study conducted by González-Domínguez et al., children with obesity had lower ferroprotein levels, with similar trends observed for total iron content, although these did not reach statistical significance. These alterations were detected only in subjects with insulin resistance, whereas children with metabolically healthy obesity exhibited an iron status similar to that of controls [43]. The findings of Ortiz-Marron et al. indicate that children with high levels of serum iron and transferrin saturation tend to have a better glycemic profile, while those with elevated transferrin concentrations exhibit a less favorable glycemic profile [44]. Lee et al. investigated the connection between indicators of body iron status and insulin resistance in Korean children, showing similar findings [45]. These two studies suggest that functional iron deficiency, indicated by low serum iron and low transferrin saturation, is linked to impaired glucose metabolism and a higher risk of insulin resistance in children [44,45]. A systematic review and meta-analysis consistently found an increased risk of ID in children and adolescents (<18 years) with obesity. However, a stratified analysis indicated that the prevalence of ID was not elevated in individuals with obesity when the diagnosis of ID was based on serum ferritin levels in the same meta-analysis. This discrepancy may be attributed to low-grade chronic inflammation [18]. On the other hand, a large, well-adjusted multicohort study confirmed a positive correlation between elevated serum ferritin and the incidence of type 2 diabetes, with this association being notably stronger in individuals without overweight or obesity [46]. A recent meta-analysis also affirmed the presence of this link in older patients [12]. The study by Wei et al. concluded that serum transferrin and sTfR levels were significantly correlated with glucose parameters. This finding suggests that transferrin and sTfR levels should be considered when investigating insulin resistance [47]. Future studies should investigate the impact of micronutrient supplementation on preventing obesity and its associated comorbidities at this age.

Table 1 reports the key studies that provide evidence of the association between ID and insulin resistance in pediatric obesity.

## 4. Interaction Between Iron Metabolism and Insulin Resistance

Iron metabolism’s significant role in the development of IR has been demonstrated in both adults and children. Studies have shown that several genetic variants of genes that regulate iron homeostasis are associated with the risk of developing diabetes and that the expression of some transcripts representing iron homeostasis genes in adipose tissue is correlated with insulin sensitivity [48,49].

A complex interaction among various tissues, such as macrophages, the skeleton, adipose tissue, liver, and muscle, may contribute to the development of insulin resistance as a result of disruptions in iron homeostasis.

High body iron stores, particularly in the liver, where it disrupts insulin signaling pathways, increase oxidative stress, and impair insulin’s ability to function effectively. Excess iron in tissues promotes the generation of reactive oxygen species (ROS), which can activate inflammatory pathways that interfere with glucose uptake [48,50,51]. High iron levels can affect adipocyte differentiation and lipid metabolism, which are essential for maintaining insulin sensitivity. They also stimulate macrophage inflammation, thereby decreasing adiponectin levels, a hormone that promotes insulin sensitivity [48,52,53]. Moreover, the iron-induced suppression of osteocalcin secretion from the skeleton also affects the secretion of adiponectin from adipose tissue [48]. The relationship between iron and insulin resistance is bidirectional, with hyperinsulinemia contributing to increased iron accumulation, creating a vicious cycle; elevated insulin levels stimulate the synthesis of ferritin, which may facilitate iron retention in various tissues and suppress hepcidin, leading to increased iron absorption and retention, further exacerbating iron overload [50,51,52,54].

Beyond iron overload, ID also contributes to the development of IR. ID could lead to the development of type 2 diabetes mellitus by impairing mitochondrial function, leading to decreased oxidative metabolism and increased reliance on anaerobic pathways, which can disrupt glucose homeostasis and enhance IR [48,53]. Moreover, ID could cause an overexpression of hepcidin, affecting glucose homeostasis [55].

In adults, the positive correlation between iron overload and insulin resistance has been proven both within the normal range of tissue iron levels and in cases of pathologic iron overload [56,57]. According to several studies, high ferritin levels, indicative of increased body iron stores, are associated with elevated insulin resistance and a higher risk of developing type 2 diabetes (T2DM) [54,58,59,60,61]. A systematic review showed that the transferrin receptor to ferritin ratio was negatively related to the risk of T2DM and that serum transferrin may be related to the development of diabetes, either directly or indirectly [62]. However, there are some contrasting data. Ko et al. showed that the positive association between ferritin and insulin resistance was present only among women with obesity, while among women without obesity, insulin resistance and the risk of diabetes were not significantly different between the high and low ferritin groups [63]. In a study on women with polycystic ovary syndrome, it has been demonstrated that insulin resistance decreases the concentration of transferrin in circulation, but does not affect the remaining parameters of iron metabolism [64]. Bahaaeldin et al. found that neither HOMA-IR nor body weight had a significant correlation with iron status markers in 90 adult diabetic patients [65]. In some studies, levels of transferrin saturation were inversely associated with T2DM [48,53,57,66]. According to Krisai’s study, lower plasma levels of available iron, rather than excessive body iron stores, could be linked to glucose dysregulation [67].

The few studies on the relationship between iron metabolism and IR conducted in children and adolescents, as mentioned in the previous paragraphs, suggest that iron dysregulation may be similarly influential in pediatric populations as in adults. However, not all studies in children and adolescents have found consistent associations between iron markers and insulin resistance. Some research has shown no significant correlation between ferritin levels and HOMA-IR in children, possibly due to the complexity of iron metabolism in growing individuals or differences in measurement techniques [68]. The contradictory findings on the correlation between iron metabolism and IR in the pediatric population may suggest that the interaction between iron status and insulin sensitivity may depend on the balance between ID and iron excess. This highlights the need for further longitudinal studies in larger and more diverse populations to better understand how iron metabolism influences insulin resistance during childhood and adolescence.

**Figure 2:**
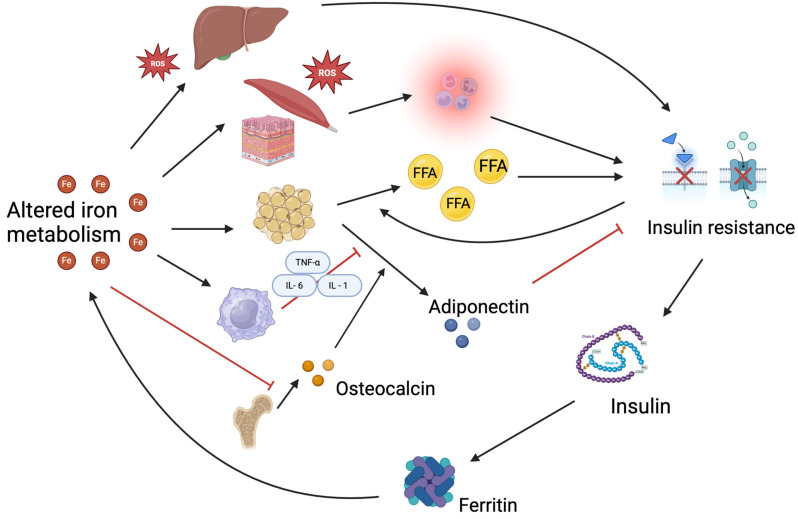
Iron metabolism and IR are linked by a complex interaction among tissues such as macrophages, the skeleton, adipose tissue, liver, and muscle. High body iron levels in the liver impair insulin signaling pathways, increase oxidative stress, and hinder insulin function. Excess iron in tissues promotes the production of reactive oxygen species (ROS), which activate inflammatory pathways that disrupt glucose uptake. Elevated iron levels can interfere with adipocyte differentiation and lipid metabolism, reducing adiponectin levels. They also promote macrophage inflammation and suppress osteocalcin secretion, which affects adiponectin secretion from adipose tissue. The relationship between iron and insulin resistance is bidirectional: hyperinsulinemia increases iron accumulation, creating a vicious cycle where elevated insulin stimulates ferritin production, leading to iron retention and decreased hepcidin levels, which, in turn, increases iron absorption and retention, worsening iron overload. FFA: free fatty acids.

In Figure 2, the complex interaction between iron metabolism and IR is schematized.

## 5. Iron Requirement and Intake in the Pediatric Population

During childhood, specifically during infancy and adolescence, iron requirements increase due to growth spurts, greater volume of blood and muscle mass, and menstrual blood losses in the case of female adolescents. Body iron stores almost double between 6 months and 1 year of age and then double again between 1 and 6 years [69]. Therefore, children and adolescents are at risk of developing iron deficiency and ID anemia. Among this population, females of reproductive age experience a greater risk of anemia due to menstrual loss [70].

Table 2 summarizes iron recommendations for females and males aged 2–17 years, based on national and international guidelines [69,70,71]. The “Reference Intake Levels for Nutrients and Energy” [71] presents the most recent version of the national (Italian) guidelines, while the European Food Safety Authority (EFSA) [72] and World Health Organization (WHO) [69] present, respectively, the European and international reference values for iron intake. Iron intake recommendations are usually expressed as “Average requirement” (AR) or “Population Reference Intake” (PRI). According to data from the Italian IV SCAI 2017–2020 Survey [73], the average iron intake was 6 mg/die in children aged 1–6 years, 7.5 mg/die in children aged 7–10 years, and 11.4 mg/die in adolescents aged 11–17 years. The average iron intakes published by EFSA are slightly higher [72]. Average iron intake ranged between 2.6 and 6.0 mg/day in infants aged < 1 year, between 5.0 and 7.0 mg/day in children aged 1 to <3 years, between 7.5 and 11.5 mg/day in children aged 3 to <10 years, between 9.2 and 14.7 mg/day in children aged 10 to <18 years. Average daily intakes were, in most cases, slightly higher in males than in females, mainly due to larger quantities of food consumed per day [72].

## 6. Iron Intake in Children with Obesity

Although obesity is related to a condition of nutritional excess, it does not exclude the presence of micronutrient deficiencies [17], among which one of the most relevant is ID [10]. Mechanisms hypothesized for this deficiency include (i) poor nutritional intake, (ii) increased iron requirements due to elevated blood volume, and (iii) a reduction in iron absorption due to enhanced inflammation [10].

Regarding dietary iron intake, Hutchinson et al., in their review [12], reported that poorer iron status in children and adolescents with overweight and obesity was independent of iron intake, which was similar to or even higher [74] in children with excess body weight than their normal-weight peers [11,41,75]. No difference occurs in dietary iron bioavailability [76,77].

Likewise, most studies have reported similar intakes of other nutrients, such as meat sources of iron, calcium (a potential inhibitor of non-heme iron absorption), and vitamin C (the most potent enhancer of non-heme iron absorption), in children with obesity compared to normal-weight children [16]. Contrary to this result, although Ferrari et al. [35] confirmed that adolescents with overweight/obesity do not differ in their estimated iron intake (total, heme, and non-heme iron), they also reported a lower intake of vitamin C in adolescents with overweight/obesity compared to normal-weight male adolescents, although this difference was not statistically significant.

The literature is still not robust regarding iron intake in children/adolescents affected by overweight or obesity. Indeed, a recent meta-analysis [13] concluded that children living with obesity have an inadequate intake of an iron-rich diet and consume high-calorie junk food. Moreover, this dietary pattern has been associated with a greater risk of nutritional deficiencies, such as iron [13].

Malden et al. [12] reviewed studies suggesting that childhood obesity may double the likelihood of developing ID, potentially due to dietary factors. Epidemiological research indicates that children with obesity tend to consume fewer iron-rich foods than those with normal weight, which could explain this connection.

The conclusions of the analysis by Kirti and Singh [78] highlight that ID is present in many dietary clusters among Indian adolescents, and it is not independent of dietary habits. Patterns characterized by high protein but low fiber and/or excessive consumption of processed foods are associated with a higher risk of ID [78].

Again, regarding diet quality, Queiroz et al. [79] focused on the consumption of ultra-processed foods (UPFs) and their association with dietary iron availability, anemia, and excess body weight in socially vulnerable children living in Brazilian slums. The study found that a higher caloric intake from UPFs negatively affects dietary iron availability and increases the risk of anemia by up to 2.5 times [79]. This situation can result from both the reduced quantity and quality of iron consumed, as well as the presence of food additives in UPFs, which can hinder nutrient digestion and absorption [79].

In conclusion, these findings indicate that ID in children and adolescents with obesity may be partly attributed to their diet.

There is agreement that inadequate iron ingestion alone does not explain the relationship between childhood obesity and ID [80]. Indeed, even if a diet is characterized by iron-rich foods, individuals with overweight or obesity could still experience deficiencies due to iron malabsorption induced by obesity-induced low-grade inflammation [81]. Additionally, individuals with obesity have increased iron requirements (secondary to increased blood volume and basal losses that accompany greater body weight) [81].

In childhood and adolescence, obesity ID connected to increased demand could be explained by increased growth and development, as well as bodily changes like the attainment of menarche during this period [81].

## 7. Nutritional and Lifestyle Strategies to Improve Iron Status in Children with Obesity

### 7.1. Effect of Weight Loss Through Balanced Diet and Physical Activity on Iron Status

The traditional way to treat ID is by providing iron supplementation [82]. However, treating ID in children with overweight poses a challenge. In this review, the studies analyzed have considered only oral iron supplements. Several studies have shown that iron supplementation is notably less effective in individuals with overweight or obesity compared to those with normal body weight [83]. This may be due to decreased iron absorption linked to elevated serum hepcidin levels, which are commonly observed in individuals with overweight or obesity [83].

This finding has also been confirmed in children with overweight [33], in whom iron supplementation therapy was not effective in restoring iron status [84,85]. This provides additional evidence of the crucial role of inflammation and serum hepcidin in iron deficiency associated with childhood obesity [86].

However, the study by Dorsey et al. [87] provides a contrasting perspective. Unexpectedly, the authors found that higher Body Mass Index (BMI) z-scores and waist-to-height ratio (WHtR) were associated with a greater likelihood of responding to iron supplementation. Interestingly, children with high BMI z-scores responded well regardless of inflammation levels, whereas those with intermediate BMI and high inflammation had the lowest probability of improvement. These findings suggest that adiposity may not universally impair iron metabolism and that a more complex interaction between body composition and inflammation should be considered [87].

Routine clinical practice of providing oral iron supplementation alone may not be effective for younger patients affected by obesity. In contrast, weight loss may improve hypoferremia [83]. A reduction in adipose tissue leads to a decrease in hepcidin release, thus increasing duodenal iron absorption and improving iron status [88].

Several studies have explored the impact of weight loss on iron status in children and adolescents with overweight or obesity. Amato et al. [88] reported a significant decrease in serum hepcidin levels and an increase in iron absorption following a 6-month weight loss program in children with obesity, resulting in an improvement in iron status. Similarly, Gong et al. [89], demonstrated that weight loss was associated with improved iron status in children aged 7–11 years who participated in a weight loss program based on nutritional education and physical activity. The review by Hutchinson et al. [16] analyzed a few intervention studies, confirming that weight loss in children and adolescents could improve iron absorption and result in an improvement in inflammatory status and indicators of iron status (e.g., serum iron and serum ferritin, hemoglobin concentrations, transferrin saturation, and serum soluble transferrin receptor (sTfR)). The same conclusions were drawn by Pande et al. [76], who underlined that lifestyle management through regular exercise and a balanced diet (reducing excessive intake of simple sugars and fats while including whole cereals, pulses, vegetables, seasonal fruits, and dry fruits) leads to beneficial restoration of both healthy body weight and iron levels. Coimbra et al. [80] showed that 5 h a week of moderate to vigorous aerobic exercise is an efficient way to reduce BMI z-scores and adiposity, improve inflammation, and consequently improve the iron profile.

An unbalanced diet, whether excessive or insufficient, may affect weight status and the serum iron profile. Some research has explored the role of a balanced diet in the prevention and management of ID in children and adolescents with overweight and obesity. Ozcelik-Ersu et al. [90] found that a higher intake of animal proteins was linked to a slightly reduced iron-binding capacity, which could indicate improved iron availability. Additionally, a greater proportion of total dietary protein was associated with slightly higher iron levels [90]. These findings highlight the need for targeted interventions that promote balanced, iron-rich dietary patterns in children participating in a personalized weight loss program, as dietary restrictions may also exacerbate anemia [91]. In fact, anemia has been found to be more prevalent in girls with obesity who were following a diet compared to those in the obesity, no diet group and the control group [91]. Even if the results are not deeply analyzed by the authors, anemia in these girls could be explained by both the restricted, unbalanced diet and the obesity-induced inflammatory status. Regardless, care should be taken when prescribing a diet to children and adolescents with obesity. The consumption of iron-rich foods should be ensured, in addition to those that enhance iron absorption. On the other hand, empty calories from snacks and sugary drinks should be limited [92], so the consumption of UPF should be reduced [79].

Lastly, studies have suggested that incorporating comprehensive parental education into the nutrition strategy for children undergoing weight loss programs could be beneficial for improving their nutritional status [93]. Equipping parents with the right knowledge and tools can help them make informed choices about their children’s diets and overall health.

### 7.2. Role of Breakfast

A strategy that may improve iron status in children is eating breakfast. The study by Lazarou and Matalas [94] showed that children who are regular breakfast eaters have higher serum iron levels and lower BMI, waist circumference, and body fat percentage compared to children who skip breakfast. The relationship between iron status and the regular habit of having breakfast was no longer significant after adjusting for physical activity, suggesting that both diet and lifestyle factors likely influence iron status [94]. In addition to breakfast, the authors also reported lower ferritin levels in adolescents who had a regular habit of skipping at least one meal per day. Similarly, Cheung et al. [95] showed that skipping breakfast was significantly associated with lower serum ferritin levels in adolescents, both with and without obesity. These results emphasize the importance of regular breakfast consumption, as it offers a key opportunity to intake essential micronutrients, particularly vitamins and minerals, that may be less available in other meals throughout the day.

Table 3 summarizes the key studies that investigate nutritional strategies to improve iron status in children and adolescents with obesity.

## 8. Limitations

We acknowledge several limitations in this review. First, it is a narrative review, providing a non-systematic summary and analysis of the existing literature on a specific topic. The absence of formal guidelines for conducting narrative reviews may introduce selection bias and often leads to qualitative rather than quantitative syntheses. For example, our review relies solely on articles available in PubMed and Scopus, potentially overlooking relevant studies indexed in other databases or search engines.

Additionally, the literature reviewed in this study provides considerable evidence that iron status in children and adolescents with obesity is largely independent of dietary iron intake. However, there is still no clear consensus on this issue. This lack of agreement may stem from the complex mechanisms underlying ID in obesity, which involve not only dietary factors but also inflammatory status, impaired iron absorption, and increased iron requirements.

Finally, while this review compiles a broad range of studies, the methodological quality of the included literature varies. Many cited studies are observational in nature, including cross-sectional and cohort designs, which limit causal inference regarding the relationship between iron metabolism, insulin resistance, and obesity in pediatric populations. Furthermore, several studies rely on self-reported dietary intake and indirect biomarkers of iron status, which are subject to recall bias and measurement variability. Another important limitation is the heterogeneity in sample sizes, with some studies including small cohorts that may not be representative of broader pediatric populations. The lack of standardization in defining key parameters, such as iron deficiency, insulin resistance, anemia, and obesity classifications, as well as variations in the methods used to assess dietary habits and nutrient intake, introduces potential inconsistencies across studies. Moreover, while we discuss associations between these factors, confounding variables, such as genetic predisposition, socioeconomic status, and inflammatory status, may contribute to the observed relationships and were not consistently controlled for in all studies.

To strengthen future research, we recommend larger, well-controlled longitudinal studies and randomized controlled trials to better delineate causal relationships and mitigate potential sources of bias.

## 9. Conclusions

The interplay among iron metabolism, IR, and obesity in children and adolescents represents a significant public health concern. Our review highlights the well-documented link between obesity-related chronic inflammation, IR, and ID, emphasizing the bidirectional relationship between iron homeostasis and metabolic health. While dietary factors alone do not fully explain ID in pediatric obesity, poor dietary habits and the consumption of ultra-processed foods warrant further study.

Interventions aimed at reducing obesity-induced inflammation, such as weight loss and physical activity, may play a crucial role in restoring iron homeostasis. Clinicians must be conscious that the best treatment for obesity-related iron deficiency may not be oral iron supplementation alone, but rather a combination with personalized weight loss. Future randomized controlled trials should compare the efficacy of iron supplementation versus integrated weight management strategies to establish optimal treatment protocols for children with obesity.

Preventive strategies should focus on early-life interventions to curb both obesity and ID. Public health initiatives, including food fortification and health education programs, have been effective in countries like China [97] and Jordan [93], but adaptation is required for children with obesity. School-based nutrition and physical activity programs, alongside parental education and community-level interventions, should be reinforced to improve pediatric health.

Further research is needed to explore the complex molecular mechanisms linking iron metabolism and IR. Longitudinal studies on the effects of weight loss and iron-related biomarkers in metabolic disease risk assessment will refine clinical guidelines. Addressing ID in children with obesity requires a comprehensive, multidisciplinary approach that integrates dietary, lifestyle, clinical, and policy interventions to enhance metabolic health outcomes.

## Figures and Tables

**Table 1:** Main studies demonstrating the link between iron deficiency and insulin resistance in pediatric population with obesity.

First Author’s Name	Type of Study	Country	Sample	Main Results
Garcìa O. P. et al. [34]	Cross-sectional study	Mexico	197, aged 6–10.5 years	Low iron concentrations in children with overweight and obesity are associated with increased levels of lipids, inflammation, and insulin resistance
H. Lee H. et al. [45]	Cross-sectional study	Korea	1350, aged 7–13 years	Iron-related factors may involve insulin resistance.
Ferrari M. et al. [35]	Cross-sectional study	Europe	876, aged 12.5–17.5 years	The level of adiposity in European adolescents was sufficient to induce chronic inflammation but did not reach the threshold necessary to compromise iron status or lead to iron deficiency.
Wei J. et al. [47]	Cross-sectional study	China	689, aged 6–18 years	Serum transferrin and sTfR were statistically significantly associated with glucose parameters.
Suàrez Ortegòn M.F. et al. [40]	Cohort study	Chile	1892, aged 4 months	Ferritin levels in infancy are positively and longitudinally associated with cardiometabolic risk at adolescent stage.
Klisic A. et al. [39]	Case–control study	Montenegro	60, aged 16–19 years	sTfR levels independently correlate with HOMA-IR; elevated ferritin and adipokines are linked to higher HOMA-IR.
Gonzàlez Dominguez A. et al. [43]	Case–control study	Spain	72, aged 6–10 years	Metal-related abnormalities were sharpened in subjects presenting IR compared to children with metabolically healthy obesity.
Ortiz Marròn H. et al. [44]	Cross-sectional study	Spain	1954, aged 9–10 years	The glycemic profile is better in children who have high concentrations of Is and transferrin saturation.

**Table 2:** Iron recommendation for females and males, aged 2–17 years, according to LARN [71], EFSA [72], and the WHO [69]. Reference intakes are expressed as mg/day.

**Female**	**LARN**	**7–12 months**	**1–3 years**	**4–6 years**	**7–10 years**	**11–14 years**		**15–17 years**
		7 (AR), 11 (PRI)	4 (AR), 8 (PRI)	5 (AR), 11 (PRI)	5 (AR), 13 (PRI)	7 (AR) 10 (PRI) *		10 (AR) 18 (PRI)
						
	**EFSA**	**7–11 months**	**1–3 years**	**4–6 years**	**7–11 years**		**12–17 years**	
		8 (AR), 11 (PRI)	5 (AR), 7 (PRI)	5 (AR), 7 (PRI)	8 (AR), 11 (PRI)		7 (AR), 13 (PRI)	
							
	**FAO/WHO** (for a dietary iron bioavailability of 15%)	**5–12 months**	**1–3 years**	**4–6 years**	**7–10 years**	**11–14 years**		**15–17 years**
		6.2 (PRI)	3.9 (PRI)	4.2 (PRI)	5.9 (PRI)	9.3 (PRI) **		20.7 (PRI)
								
**Male**	**LARN**	**7–12 months**	**1–3 years**	**4–6 years**	**7–10 years**	**11–14 years**		**15–17 years**
		7 (AR), 11 (PRI)	4 (AR), 8 (PRI)	5 (AR), 11 (PRI)	5 (AR), 13 (PRI)	7 (AR) 10 (PRI)		9 (AR) 13 (PRI)
							
	**EFSA**	**7–11 months**	**1–3 years**	**4–6 years**	**7–11 years**		**12–17 years**	
		8 (AR), 11 (PRI)	5 (AR), 7 (PRI)	5 (AR), 7 (PRI)	8 (AR), 11 (PRI)		8 (AR), 11 (PRI)	
							
	**FAO/WHO** (for a dietary iron bioavailability of 15%)	**7–12 months**	**1–3 years**	**4–6 years**	**7–10 years**	**11–14 years**		**15–17 years**
		6.2 (PRI)	3.9 (PRI)	4.2 (PRI)	5.9 (PRI)	9.7 (PRI)		12.5 (PRI)

* 10 (AR), 18 (PRI) if menstruation; ** 21.8 (PRI) if menstruation. **Abbreviations:** AR = Average requirement; PRI = Population Reference Intake; EFSA = European Food Safety Authority; FAO = Food and Agriculture Organization; LARN = Livelli di assunzione di riferimento di nutrienti ed energia per la popolazione italiana; WHO = World Health Organization.

**Table 3:** Main studies on nutritional strategies to improve iron status in children and adolescents with obesity.

First Author’s Name	Type of Study	Sample	Objectives	Main Results
Association of iron deficiency with dietary parameters or habits				
Ferrari M. et al. [35]	Cross-sectional study	876 adolescents aged 12.5–17.5 years	To investigate the association among obesity, inflammation, and iron status. To assess the intake of relevant nutrients and their association with BMI z-score, FM, and FFM.	No significant (*p* > 0.05) differences between BMI and the intake of: Total iron g/day (♂ normal weight 15.6 ± 0.4 vs. ♂ overweight 14 ± 0.8; ♀ normal weight 12 ± 0.2 vs. ♀ overweight 11.7 ± 0.5); Heme iron g/day (♂ normal weight 2.1 ± 0.1 vs. ♂ overweight 1.9 ± 0.2; ♀ normal weight 1.5 ± 0.2 vs. ♀ overweight 1.6 ± 0.2); Non-heme iron (♂ normal weight 13.4 ± 0.4 vs. ♂ overweight 12.3 ± 0.7; ♀ normal weight 10.6 ± 0.2 vs. ♀ overweight 10.2 ± 0.5).
Kirti K. et al. [78]	Cross-sectional study	12,318 adolescents aged 10–19 years	To identify clusters based on adolescents’ dietary patterns; to correlate clusters with obesity prevalence, lipid anomalies, hypertension, and micronutrient deficiencies	Five disjointed clusters were described based on individuals’ consumption of 17 dietary items. ID is not independent of dietary habits, as was observed in every cluster and is most prevalent in the “Plant based” (17.72; SD = 0.79; *p* < 0.001), “Western” (17.69; SD = 0.87; *p* = 0.013), and “Obesogenic diet” (17.65; SD = 0.74; *p* = 0.330) clusters, while the “Convenient” (14.27; SD = 0.75; *p* = 0.017) and “Comparatively healthy” (12.79; SD = 0.74; *p* < 0.001) clusters exhibited lower prevalence. Low fiber intake and/or excessive consumption of processed foods are associated with a higher risk of ID.
Ozcelik-Ersu D. et al. [90]	Cross-sectional study	93 children and adolescents with obesity aged 10–17 years	To examine the relationship between nutritional status and biochemical parameters	Negative weak correlation between dietary animal-source protein consumption and total protein percentage and iron-binding capacity (r = −0.282, *p* = 0.006 and r = −0.265, *p* = 0.010, respectively). Positive weak correlation between total protein percentage and serum iron levels (r = 0.229, *p* = 0.027).
Queiroz J. et al. [79]	Cross-sectional study	443 children aged 6–59 months	To assess iron availability and the presence of anemia and excess body weight and to determine their association with ultra-processed food (UPF) consumption.	The highest relative share of UPF in total calorie consumption is inversely associated with iron availability (β quartile 4 versus quartile 1: −0.12; 95% CI: −0.23; −0.01; *p* = 0.037); and directly associated with excess body weight (OR quartile 4 versus quartile 1: 2.16; 95% CI 1.05; 4.46; *p* = 0.038) and anemia (OR quartile 4 versus quartile 1: 2.45; 95% CI: 1.26; 4.78; *p* = 0.009).
Yıldırım O. et al. [91]	Cross-sectional study	Adolescents with obesity aged 12–19 years, divided into 2 groups: N = 29 anemics, N = 33 non-anemics; there was also a third control group of 33 healthy individuals without obesity	To assess the effect of anemia (defined as Hb ≤ 12 g/dL in women and ≤13 g/dL in men) on cardiovascular findings in adolescents with obesity	In the anemic group compared to the non-anemic group, ferritin was significantly lower (18.9 ± 14.9 versus 28.2 ± 15.8 ng/mL, *p* = 0.023), while CRP was significantly higher (8.3 ± 8.4 versus 3.4 ± 4.0 mg/dL, *p* = 0.002). 28% of the anemic patients were on a diet, versus 3% of non-anemic patients and 3% of the control group (*p* = 0.020).
Nutritional and lifestyle strategy				
Cheung Y.T. et al. [95]	Cross-sectional study	523 adolescents aged 16–19 years	To determine the prevalence of ID and IDA. To identify the dietary predictors of iron status. To evaluate the association between iron status and functional outcomes (HRQoL and fatigue).	The overall prevalence of ID was 11.1%, and 10.9% of the girls had IDA. 36.3% reported a regular habit of skipping ≥1 meal/day. Lower ferritin in adolescents who skipped meals (Est = −35.1, *p* = 0.017).
Coimbra S. et al. [80]	Longitudinal intervention study	73 children and adolescents aged 5–17 years; intervention group: N = 44, control group: N = 29	To evaluate the impact of an 8-month school-based physical exercise program on hepcidin levels, inflammation markers, and iron metabolism.	The PE group showed a decrease in BMI z-score (*p* = 0.003), body fat mass (*p* = 0.012), CRP (*p* = 0.002), IL-6 (*p* = 0.048), ferritin (*p* = 0.013), hepcidin (*p* = 0.040), and sTfR (*p* = 0.010), as well as an increase in iron concentration (*p* = 0.002).
Dorsey A.F. et al. [87]	Intervention study	50 children aged 2–5 years	To test 1-month iron supplementation (15 mg/day) in children with anemia (Hb < 11 g/dL), also testing the following conditions: (a) immune activation (CRP) is associated with a lack of response to iron supplementation; (b) body fat (BMI, WHtR) moderates the association between immune function and response to treatment.	50% of children “responders” to supplementation (HB ≥ 11 g/dL) High CRP associated with lower response to supplementation (OR: 0.19, CI: 0.03–1.08, *p*-value < 0.10) Higher WHtR (>0.5) (OR: 32.54, CI: 2.67–396.08, *p*-value: <0.05) and higher BMI z-score (>1.0) (OR: 3.76, CI: 0.96–14.79, *p*-value: <0.10) were associated with response to supplementation
Lazarou C. et al. [94]	Cross-sectional study	83 children aged 6–12 years	To assess the association between breakfast intake and Mediterranean diet adherence (evaluated with modified KIDMED score), physical activity levels, obesity, selected cardiovascular risk markers, and iron status.	Breakfast skippers were 14% more likely to have a body fat percentage higher by 1 unit, as well as higher values for both BMI and waist circumference. Serum iron was higher for breakfast eaters (mean value 82.79 ± 29.71 μg/dL), compared to breakfast skippers (69.97 ± 27.97 μg/dL, *p =* 0.050). No differences in the mean values of either ferritin (41.36 vs. 41.22 ng/dL) or Hb (13.82 vs. 13.49 g/dL).
Reviews				
Alshwaiyat N. et al. [83]	Review	Experimental article on the relationship between ID and obesity conducted from January 2015 to January 2021 focusing on individuals with overweight and obesity (children/adolescents and adults)	To discuss the evidence on the relationship between obesity and iron deficiency	Obesity can disrupt iron balance, leading to IDA, potentially due to inflammation-driven increases in hepcidin levels. Weight loss helps reduce inflammation and hepcidin, thereby enhancing iron absorption and improving iron status.
Berton P.F. et al. [11]	Systematic review	2543 children and adolescents, aged 3–21 years, from 16 articles.	To study iron deficiency in children and adolescents with obesity and its association with inflammation (interleukins) and hepcidin.	Obesity’s chronic inflammation leads to the production of IL-6, which stimulates hepcidin synthesis, resulting in ID. ID is common in children and adolescents with obesity, who respond inadequately to iron supplementation but respond adequately to interventions against chronic inflammation, such as weight loss and physical activities.
Calcaterra V. et al. [10]	Narrative review	45 articles published from 2008 to 2023 focusing on micronutrient deficiencies in childhood obesity	To analyze and summarize main deficiencies associated with obesity, their clinical consequences, and evidence regarding possible supplementation	Iron is one of the most common deficient microelements (together with vitamins A, B, C, D, and E; folic acid; zinc; and copper). Relationship between obesity and micronutrient deficiencies remains unclear. Weight loss was linked to improved iron and inflammatory status.
Feldman A. et al. [96]	Narrative review	Findings from pediatric studies. Relevant data from adult studies are included when applicable for clinical extrapolation	To summarize available data about iron and copper in the context of obesity and NAFLD in children.	Perturbations of iron homeostasis shown to contribute to the pathogenesis of NAFLD (not sufficiently examined in pediatric cohorts). Iron supplementation is less effective in children with overweight. Weight reduction leads to a decrease in hepcidin and leptin and to an increase in iron absorption and an improvement of iron status.
Grandone A. et al. [25]	Narrative Review	Studies on children; Studies on adults to support pathophysiological aspects	To study ID in children with obesity and the role of hepcidin, as well as iron status and its consequences on health, particularly regarding cognitive function and and obesity	The best treatment for obesity-related ID may be weight loss, alone or in combination with iron supplementation.
Hutchinson C. [16]	Narrative Review	48 observational studies, case reports, and interventional studies published until December 2015 and conducted on children and adolescents.	To evaluate the relationship between iron and overweight and obesity in children and adolescents, with an emphasis on iron status, oral iron response, dietary intake, and inflammatory markers.	ID (or risk of ID) is more prevalent among children and adolescents with overweight and obesity; chronic inflammation is a plausible explanation, rather than dietary factors. Weight loss improves inflammatory status and indicators of iron status. Children and adolescents with overweight/obesity have reduced response to oral iron.
Ibrahim L. et al. [92]	Literature narrative review	Data from children under the age of 5 years	To review the association between ID and obesity in toddlers and preschool children.	Conflicting results, but most articles agree that ID is significantly associated with overweight and obesity in children; systemic inflammatory reaction seems to be the major cause through hepcidin, which decreases the duodenal absorption of iron, in addition to other causes, including dietary and genetic factors. Unbalanced diet, either in excess or shortage, may affect serum iron. Dietary interventions aimed at promoting a balanced diet and limiting the consumption of calorie-dense, low-nutrient foods may be beneficial.
Malden S. [12]	Systematic review and meta-analysis	9381 children aged 2–19 years (from 10 different studies focusing on iron deficiency)	To study the association between different medical conditions and comorbidities and obesity in young children.	Having obesity doubled the odds of iron deficiency diagnosis (OR 2.1; 95% CI 1.4–3.2). The condition remains associated with obesity even when controlling for diet as a covariate.
Pande S. et al. [76]	Narrative Review	Data from studies on children and adolescents with obesity. Some animal experiments and data from adult studies are also cited.	To establish the role hepcidin plays in obesity and its relation with anemia. To endorse BMI as a biomarker for anemia in adolescents with obesity.	Adolescents with obesity were twice as likely to be anemic than normal-weight adolescents; hepcidin mediates the anemia through obesity-induced inflammation. Screening for iron status among adolescents with elevated BMI is advisable. Weight reduction can be useful to reduce inflammation and improve iron absorption.
Sachdeva M. et al. [13]	Systematic review and meta-analysis	49,206 children and adolescents aged <18 years, from 42 studies	To examine the association between obesity and ID, IDA, and various hematological parameters.	Pooled OR (95% CI) for ID = 1.64 (1.22, 2.21; *p* = 0.001), and pooled prevalence of ID = 20.07% (14.98, 25.16) among children living with obesity. Pooled OR (95% CI) for IDA = 0.78 (0.43, 1.43, *p* = 0.43).
Were J. et al. [81]	Scoping review	720 studies on the coexistence of undernutrition and overnutrition among women of reproductive age (15–49 y) and preschool children (≤5 y) in low- and middle-income countries	To map the literature on the DBM, providing an understanding of how the DBM construct has been defined in the current literature. To elucidate plausible mechanisms underlying DBM development and its common risk factor.	The understanding of the DBM in the literature is ambiguous. The predominant mechanism that emerged with regard to overweight/obesity and ID DBM was chronic low-grade inflammation.

**Abbreviations:** ID, iron deficiency; IDA, iron deficiency anemia; Hb, Hemoglobin; CRP, c-reactive protein; IL-6, interleukin-6; sTfR, soluble transferrin receptor; BMI, Body Mass Index; WHtR, waist-to-height ratio; FM, Fat Mass; FFM, Fat Free Mass; DBM, Double Burden of Malnutrition; NAFLD, Nonalcoholic Fatty Liver Disease; UPF, Ultra-Processed Foods; HRQoL, Health Related Quality of Life.
